# Association between dietary copper, iron, zinc, selenium intake and osteopenia or osteoporosis in elderly hypertensive patients: a retrospective cohort study

**DOI:** 10.3389/fnut.2024.1419379

**Published:** 2024-08-14

**Authors:** Mingji Chen, Long Jia, Rufeng Gao

**Affiliations:** Department of Orthopaedics, Qingpu Branch of Zhongshan Hospital Affiliated to Fudan University, Shanghai, China

**Keywords:** osteoporosis, hypertensive, iron, zinc, copper, selenium

## Abstract

**Aim:**

The study aimed to investigate the link between dietary copper, iron, zinc, selenium intake with osteopenia and osteoporosis in elderly hypertensive patients.

**Methods:**

The data of hypertensive patients were extracted from the National Health and Nutrition Examination Survey 2005–2010, 2013–2014, and 2017–2018. Data of dietary iron, zinc, copper and selenium intakes were obtained according to 24-h diet recall interviews. Osteopenia and osteoporosis were determined based on the bone mineral density. Weighted liner regression and weighted logistic regression were employed to assess the association between iron, zinc, copper, and selenium intakes with osteopenia and osteoporosis. All results were presented as β, odds ratios (ORs), and 95% confidence intervals (CIs).

**Results:**

In total, 5,286 elderly hypertensive patients were included. Among them, 2,961 (56.02%) patients have osteopenia, and 566 (10.71%) have osteoporosis. After adjusting all covariates, dietary copper intake ≥the recommended daily allowance was positively correlated with bone mineral density on total femur (β = 0.086, 95% CI: 0.021–0.152) and femoral neck (β = 0.097, 95% CI: 0.016–0.178). Dietary zinc intake ≥the recommended daily allowance was also positively correlated with bone mineral density on total femur (β = 0.092, 95% CI: 0.030–0.153) and femoral neck (β = 0.122, 95% CI: 0.050–0.193). Dietary copper (O = 0.581, 95% CI: 0.394–0.858) and zinc (OR = 0.595, 95% CI: 0.429–0.827) intake ≥the recommended daily allowance levels were related to increased odds of osteoporosis in elderly with hypertension.

**Conclusion:**

Higher dietary copper and zinc intake was associated with lower odds of osteoporosis in the elderly hypertensive patients. Higher dietary intake included copper and zinc may be beneficial for the bone health in the elderly hypertensive patients.

## Introduction

Osteopenia and osteoporosis are prevalent conditions among the elderly population, characterized by reduced bone mineral density (BMD) and increased fracture risk. Reduced bone density heightens the fractures risk, leading to increased healthcare expense, physical disability, diminished quality of life, and increased mortality risk, placing huge burden on individuals and society (1, 2). Previous study has reported hypertension was independently associated with osteoporosis in the general population (3). The patients with osteoporosis is usually affected by hypertension (4). Variations of the blood pressure might increase the risk of fall thus it increases the risk of osteoporotic fracture (5). The co-existence of the history of fractures and hypertension could increase the all-cause death risk of osteoporosis (6).

Dietary components have been identified as crucial elements that influence bone health (7). Iron, zinc, copper and selenium are essential trace elements for human wellbeing, in the absence of appropriate levels, can lead to chronic inflammatory state of the body (8–11). And chronic inflammatory also plays significant role in the development and progression of hypertension and reduced BMD (12, 13). Reduced intracellular iron levels can cause problem with the functioning and activity of osteoblasts and osteoclasts, leading to bone loss (14). Patients with osteoporosis have low serum zinc concentrations level (15). Copper deficiency can lead to Menkes' disease, and osteoporosis is one of the main adversities consequences of Menkes' disease (16, 17). Additionally, high serum selenium concentrations associated with higher odds of fracture (18, 19).

Our study aimed to investigate the relationship between dietary copper, iron, zinc, selenium intake and osteoporosis in the elderly with hypertension.

## Materials and methods

### Study design and participants

In this retrospective cohort study, data for hypertensive patients were extracted from the National Health and Nutrition Examination Survey (NHANES) database in five survey cycles (2005–2006, 2007–2008, 2009–2010, 2013–2014, and 2017–2018). The NHANES is a study designed to assess the health and nutritional status of adults and children in the United States, comprising in-person interviews and physical examinations.

Hypertensive patients were determined of those having systolic blood pressure ≥ 130 mmHg or/and diastolic blood pressure ≥ 80 mmHg, having self-reported hypertension history, or taking antihypertensive medications (20). Hypertensive patients were included with following criteria: (1) with complete BMD information, (2) without taking bone resorption, (3) age ≥ 60 years old, (4) with complete dietary intake data on trace elements. Patients were excluded with extreme energy intake (male: < 500 or >8,000 kcal, female: < 500 or >5,000 kcal), and missing covariates data.

### Dietary trace elements intake assessment

Dietary trace elements intake was obtained from the 24-h dietary recall interviews. A trained dietary interviewer performed a face-to-face interview, with a second interview by telephone 3–10 days later to gather further information. However, only 3,057 out of 5,286 participants were available for the second dietary recall. Thus, the first 24-h dietary recall provided the dataset for our study. Dietary copper, iron, zinc, selenium intakes were categorized into less than recommended daily allowance (RDA) and equal or more than RDA categories (21).

### BMD measurements and definition of osteopenia and osteoporosis

BMD measurements were conducted using a dual X-ray absorptiometry technique with Hologic QDR-4500A fan-beam densitometers (Hologic, Inc., Bedford, MA, United States) while the participants visited mobile examination centers. The left hip was routinely scanned to report the total BMD of the femur, femoral neck, and trochanter. If the left hip had been replaced or metal objects had been injection, then the right hip was scanned instead. Osteopenia and osteoporosis were assessed using BMD. BMD data were standardized to eliminate unit bias, with the BMD values for men and women aged 20–29 serving as reference values. Osteopenia was defined as a BMD between 1 and 2.5 standard deviations below the mean for participants aged 20–29, whereas osteoporosis was defined as a BMD more than 2.5 standard deviations below the mean (22). The BMD values for the total femur, femoral neck, trochanter, and intertrochanter were all ≥1 standard deviation above the mean BMD level defined as normal.

### Covariates

Sociodemographic variables include age, gender (male and female), race (Non-Hispanic White, Non-Hispanic Black, Mexican American and other race), Income-to-poverty ratio (< 1, ≥1, unknown), education level (below high school, high school/GED or equivalent, above high school) and marriage status (married/living with partner, spinsterhood/separated/divorced/widowed). Behavioral characteristics include smoking (no, former, now), alcohol drinking (no, yes, unknown), physical activity (mild, moderate/heavy, unknown), and caffeine intake. Health factors include body mass index, chronic kidney disease, diabetes, dyslipidemia, cardiovascular disease, chronic obstructive pulmonary disease (COPD), cancer and family history of osteoporosis. Chronic kidney disease was defined as estimate glomerular filtration rate < 60 ml/min per 1.73 m^2^ or urine albumin-to-creatinine ratio ≥ 30 mg/g (23). Diabetes were determined of patients having a fasting plasma glucose level ≥ 7.0 mmol/L or Hemoglobin A1c level ≥ 6.5%, having self-reported diabetes history, or taking hypoglycemic medications (24). Cardiovascular disease was determined using self-reported physician diagnoses obtained from standardized medical condition questionnaires and cardiovascular medications history (25). Patients were considered having cardiovascular disease with a positive response to the question “Has a doctor or other health expert ever informed you that you have congestive heart failure/coronary heart disease/angina/heart strike/and stroke”. COPD patients were defined as answering “yes” to the question “Has a doctor or other health professional ever told you that you had COPD” (26). Thiazide and use of other hypotensor were also recorded. Laboratory measurements include hormones, glucocorticoid, white blood cell count, lymphocyte and neutrophil. Dietary information documents total energy intake, sodium, calcium, and Vitamin K.

### Statistical analysis

Accounting for the complex sampling design, we corrected all statistical analyses for study design and weighting variables and ensured national representativeness. Sample weighting (WTDRD1), stratification (SDMVSTRA), and primary sampling unit (SDMVPSU) were considered in the complex survey design. Quantitative data was presented as mean and standard error [Mean (S.E)], and qualitative data was expressed as frequency and percentage [*n* (%)]. Dietary copper, iron, zinc and selenium intakes were categorized based on tertiles. The potential covariates were selected using weight univariate linear regression models and logistic regression models. Weighted univariate and multivariate linear regression models and logistic regression models were used to explore the association of dietary copper, iron, zinc and selenium intake with BMD, osteopenia and osteoporosis in hypertensive patients. The results were presented as β, odds ratios (ORs), and 95% confidence intervals (CIs). All statistical analyses were conducted using R version 4.2.3 (2023-03-15 ucrt), and *P* < 0.05 were considered statistical differences.

### Ethical statement

The study was approved by the National Center for Health Statistics Ethics Review Board, and written informed consents were provided by all participants. Ethical approval for our study was waived by the ethics committee of Qingpu Branch of Zhongshan Hospital Affiliated to Fudan University.

## Results

### Characteristics of hypertensive patients

**Figure 1:**
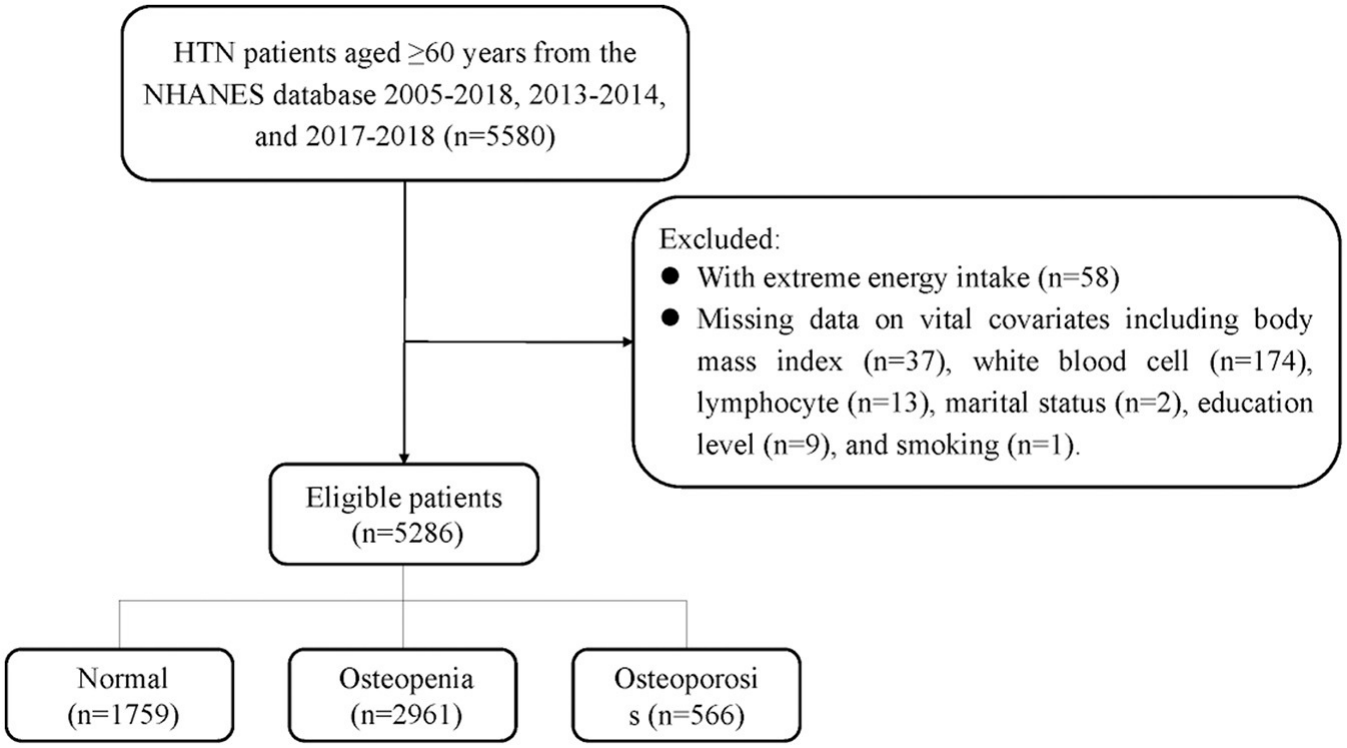
The flow chart of participant selection.

The sampling screening process was illustrated in Figure 1. A total of 15,599 patients with hypertension were initially included. After excluding patients younger than 60 years old (*n* = 5,165), taking bone resorption inhibitors (*n* = 273), having extreme energy intake (*n* = 58), missing data on BMD (*n* = 4,309), dietary trace elements (*n* = 272), and important covariates (*n* = 236). Finally, 5,286 patients were included for final analysis. The characteristics of elderly hypertensive patients were shown in Tables 1, 2. Of these patients, the mean age was 69.88 (±0.13) years old, with 2,425 (50.67%) female. 2,961 (56.02%) patients were classified into osteopenia, and 566 (10.71%) were osteoporosis. Among the three groups, there were statistical differences in variables of age, gender, race, marriage, education level, height, weight, body mass index, smoking, drinking, physical, chronic kidney disease, diabetes, dyslipidemia, COPD, cancer, family history of osteoporosis, hormones, thiazide, other hypotensor, total energy intake, Na, Ca, BMD of total femur and femoral neck, and dietary intake of copper, iron, zinc, selenium (all *P* < 0.05).

**Table 1:** Characteristics of elderly hypertensive patients.

**Variables**	**Total (*N* = 5,286)**	**Normal (*N* = 1,759)**	**Osteopenia (*N* = 2,961)**	**Osteoporosis (*N* = 566)**	***P***
Age, years, Mean (±S.E)	69.88 (±0.13)	68.02 (±0.19)	70.11 (±0.18)	73.81 (±0.36)	< 0.001^#^
Gender, *n* (%)					< 0.001^*^
Female	2,425 (50.67)	723 (45.84)	1,362 (49.78)	340 (68.51)	
Male	2,861 (49.33)	1,036 (54.16)	1,599 (50.22)	226 (31.49)	
Race, *n* (%)					< 0.001^*^
Non-Hispanic white	2,779 (78.23)	743 (72.72)	1,646 (80.15)	390 (83.29)	
Non-Hispanic black	1,114 (9.15)	596 (15.79)	465 (6.60)	53 (4.21)	
Mexican American	663 (4.49)	217 (4.52)	397 (4.74)	49 (3.10)	
Other race	730 (8.14)	203 (6.97)	453 (8.51)	74 (9.40)	
PIR, *n* (%)					0.124^*^
< 1	720 (7.61)	220 (6.67)	417 (7.61)	83 (10.19)	
≥1	4,108 (84.79)	1,402 (86.61)	2,274 (84.35)	432 (82.08)	
Unknown	458 (7.60)	137 (6.72)	270 (8.04)	51 (7.74)	
Marriage, *n* (%)					< 0.001^*^
Married/ living with partner	3,206 (64.92)	1,148 (70.76)	1,799 (65.35)	259 (46.73)	
Spinsterhood/ separated/ divorced/ widowed	2,080 (35.08)	611 (29.24)	1,162 (34.65)	307 (53.27)	
Education, *n* (%)					0.023^*^
Below high school	1,574 (19.84)	523 (19.58)	856 (18.77)	195 (26.09)	
High school/ GED or equivalent	1,340 (26.95)	409 (25.15)	781 (27.97)	150 (26.57)	
Above high school	2,372 (53.21)	827 (55.27)	1,324 (53.26)	221 (47.34)	
Height, cm, mean (±S.E)	166.61 (±0.19)	168.87 (±0.31)	166.59 (±0.29)	160.55 (±0.50)	< 0.001^#^
Weight, kg, mean (±S.E)	80.81 (±0.37)	89.14 (±0.67)	79.12 (±0.54)	66.83 (±1.19)	< 0.001^#^
BMI, *n* (%)					< 0.001^*^
< 25	1,257 (22.35)	215 (11.20)	759 (23.56)	283 (46.55)	
25–30	2,078 (39.53)	599 (34.00)	1,281 (43.25)	198 (35.34)	
≥30	1,951 (38.12)	945 (54.80)	921 (33.19)	85 (18.12)	
Smoking, *n* (%)					< 0.001^*^
No	2,513 (48.08)	842 (48.98)	1,396 (47.14)	275 (50.51)	
Former	2,110 (40.85)	715 (42.40)	1,193 (41.67)	202 (32.29)	
Now	663 (11.07)	202 (8.62)	372 (11.19)	89 (17.20)	
Drinking, *n* (%)					< 0.001^*^
No	1,368 (23.23)	435 (22.02)	762 (22.80)	171 (28.76)	
Yes	2,568 (54.60)	889 (57.01)	1,475 (55.84)	204 (41.57)	
Unknown	1,350 (22.17)	435 (20.97)	724 (21.36)	191 (29.66)	
Physical, *n* (%)					< 0.001^*^
Mild	3,228 (57.50)	1,042 (56.76)	1,782 (55.95)	404 (67.51)	
Moderate/heavy	1,682 (35.32)	612 (37.87)	949 (36.00)	121 (24.84)	
Unknown	376 (7.18)	105 (5.36)	230 (8.04)	41 (7.65)	
CKD, *n* (%)					< 0.001^*^
Yes	1,687 (28.15)	517 (25.91)	915 (27.09)	255 (39.72)	
Diabetes, *n* (%)					< 0.001^*^
Yes	1,689 (27.32)	661 (33.40)	886 (25.29)	142 (21.23)	
Dyslipidemia, *n* (%)					0.015^*^
Yes	4,583 (88.23)	1,524 (88.60)	2,586 (89.01)	473 (83.18)	
CVD, *n* (%)					0.245^*^
Yes	2,246 (43.26)	707 (41.51)	1,256 (43.51)	283 (46.69)	
COPD, *n* (%)					0.028^*^
Yes	663 (13.00)	205 (11.71)	357 (12.71)	101 (18.02)	
Cancer, *n* (%)					0.037^*^
Yes	1,151 (25.39)	337 (22.16)	667 (26.17)	147 (30.14)	
Family history of osteoporosis, *n* (%)					0.024^*^
Yes	491 (12.62)	145 (10.62)	280 (12.57)	66 (18.42)	
Hormones, *n* (%)					< 0.001^*^
Yes	146 (4.12)	77 (5.97)	63 (3.78)	6 (0.83)	
Glucocorticoid, *n* (%)					0.499^*^
Yes	109 (2.29)	30 (2.72)	61 (2.02)	18 (2.47)	
Thiazide, *n* (%)					< 0.001^*^
Yes	612 (11.53)	248 (14.63)	330 (11.04)	34 (5.60)	
Other Hypotensor, *n* (%)					0.047^*^
Yes	3,233 (60.32)	1,138 (63.32)	1,768 (59.74)	327 (55.19)	
Energy, kcal, mean (±S.E)	1,866.45 (±18.01)	1,905.96 (±27.80)	1,883.09 (±21.93)	1,672.13 (±38.32)	< 0.001^#^
Na, mg, mean (±S.E)	3,115.57 (±32.65)	3,163.08 (±56.14)	3,153.17 (±38.99)	2,790.54 (±68.51)	< 0.001^#^
Ca, mg, mean (±S.E)	865.05 (±11.34)	897.17 (±20.09)	858.31 (±13.66)	812.28 (±23.09)	0.021^#^
Caffeine, mg, mean (±S.E)	172.82 (±3.37)	173.34 (±5.58)	175.59 (±4.17)	157.02 (±11.63)	0.320^#^
Vitamin K, mcg, mean (±S.E)	106.15 (±2.53)	107.16 (±4.28)	108.17 (±3.65)	92.96 (±7.62)	0.208^#^
WBC, 1,000/μL, mean (±S.E)	7.23 (±0.06)	7.27 (±0.08)	7.22 (±0.09)	7.17 (±0.09)	0.771^#^
Lymphocyte, 1,000/μL, mean (±S.E)	2.05 (±0.04)	2.08 (±0.04)	2.05 (±0.07)	1.95 (±0.04)	0.089^#^
Neutrophil, 1,000/μL, Mean (±S.E)	4.31 (±0.03)	4.31 (±0.07)	4.31 (±0.05)	4.34 (±0.07)	0.905^#^

S.E, standard error.

^#^Weighted analysis of variance.

^*^Rao-Scott Chi-square test.

PIR, poverty income ratio; BMI, body mass index; CKD, chronic kidney disease; CVD, cardiovascular disease; COPD, chronic obstructive pulmonary disease; Na, sodium; Ca, calcium; WBC, white blood cell.

**Table 2:** Characteristics on bone mineral density and trace element intake in hypertensive patients.

**Variables**	**Total (*N* = 5,286)**	**Normal (*N* = 1,759)**	**Osteopenia (*N* = 2,961)**	**Osteoporosis (*N* = 566)**	***P***
**BMD of specific sites**					
Total femur, gm/cm^2^, mean (±S.E)	0.91 (±0.00)	1.07 (±0.00)	0.87 (±0.00)	0.67 (±0.01)	< 0.001^#^
Femoral neck, gm/cm^2^, mean (±S.E)	0.74 (±0.00)	0.90 (±0.00)	0.70 (±0.00)	0.53 (±0.01)	< 0.001^#^
Cu, mg, mean (±S.E)	1.22 (±0.02)	1.25 (±0.03)	1.23 (±0.02)	1.02 (±0.03)	< 0.001^#^
Cu RDA, *n* (%)					< 0.002^*^
No	1,980 (34.61)	603 (31.97)	1,124 (34.17)	253 (44.12)	
Yes	3,306 (65.39)	1,156 (68.03)	1,837 (65.83)	313 (55.88)	
Fe, mg, mean (±S.E)	14.30 (±0.18)	14.20 (±0.26)	14.62 (±0.22)	12.91 (±0.37)	< 0.001^#^
Fe RDA, *n* (%)					0.009^*^
No	1,097 (19.27)	336 (18.97)	618 (18.19)	143 (25.70)	
Yes	4,189 (80.73)	1,423 (81.03)	2,343 (81.81)	423 (74.30)	
Zn, mg, mean (±S.E)	10.80 (±0.26)	10.75 (±0.22)	11.13 (±0.38)	9.22 (±0.28)	< 0.001^#^
Zn RDA, *n* (%)					0.006^*^
No	2,934 (50.35)	972 (48.41)	1,616 (49.78)	346 (58.66)	
Yes	2,352 (49.65)	787 (51.59)	1,345 (50.22)	220 (41.34)	
Se, mg, mean (±S.E)	100.62 (±1.11)	103.37 (±1.81)	101.43 (±1.30)	88.95 (±2.61)	< 0.001^#^
Se RDA, *n* (%)					< 0.003^*^
No	966 (16.78)	280 (14.60)	551 (16.72)	135 (23.08)	
Yes	4,320 (83.22)	1,479 (85.40)	2,410 (83.28)	431 (76.92)	

S.E, standard error.

^#^Weighted analysis of variance.

^*^Rao-Scott Chi-square test.

Cu, Copper; Fe, Iron; Zn, Zinc; Se, Selenium; RDA, recommended daily allowance.

### Association between trace elements intake and BMD

The associations between dietary copper, iron, zinc, selenium intake and BMD in patients with hypertension were illustrated in Table 3. After adjusting demographic characteristics, dietary copper intake (β = 0.101, 95% CI: 0.036–0.166), iron intake (β = 0.086, 95% CI: 0.011–0.161), zinc intake (β = 0.110, 95% CI: 0.055–0.164), selenium intake (β = 0.033, 95% CI: 0.001–0.064) was positively correlated with BMD on total femur in the elderly hypertensive patients. Dietary zinc intake was also positively correlated with BMD on femoral neck (β = 0.097, 95% CI: 0.032–0.162) in hypertension. After adjusting all covariates, dietary copper intake was positively correlated with BMD on total femur (β = 0.086, 95% CI: 0.021–0.152) and femoral neck (β = 0.097, 95% CI: 0.016–0.178). Dietary zinc intake was also positively correlated with BMD on total femur (β = 0.092, 95% CI: 0.030–0.153) and femoral neck (β = 0.122, 95% CI: 0.050–0.193).

**Table 3:** Association between trace element intake and bone mineral density in hypertension.

**Variables**	**Total femur**		**Femoral neck**	
	**β (95% CI)**	***P***	**β (95% CI)**	***P***
**Model 1**				
Cu	0.050 (0.003–0.098)	0.039	0.047 (−0.001 to 0.094)	0.053
Cu RDA				
No	Ref		Ref	
Yes	0.101 (0.036–0.166)	0.003	0.075 (−0.004 to 0.154)	0.064
Fe	0.029 (0.006–0.053)	0.017	0.011 (−0.017 to 0.038)	0.439
Fe RDA				
No	Ref		Ref	
Yes	0.086 (0.011–0.161)	0.026	0.041 (−0.039 to 0.121)	0.309
Zn	0.014 (−0.030 to 0.057)	0.532	0.010 (−0.028 to 0.048)	0.613
Zn RDA				
No	Ref		Ref	
Yes	0.110 (0.055–0.164)	< 0.001	0.097 (0.032–0.162)	0.004
Se	0.033 (0.001–0.064)	0.042	0.019 (−0.012 to 0.051)	0.228
Se RDA				
No	Ref		Ref	
Yes	0.057 (−0.018 to 0.131)	0.132	0.060 (−0.022 to 0.143)	0.066
**Model 2**				
Cu	0.038 (−0.008 to 0.085)	0.103	0.051 (0.002–0.099)	0.040
Cu RDA				
No	Ref		Ref	
Yes	0.086 (0.021–0.152)	0.011	0.097 (0.016–0.178)	0.019
Fe	0.022 (−0.004 to 0.048)	0.101	0.015 (−0.016 to 0.046)	0.334
Fe RDA				
No	Ref		Ref	
Yes	0.034 (−0.042 to 0.110)	0.376	0.020 (−0.063 to 0.102)	0.634
Zn	−0.002 (−0.039 to 0.035)	0.905	0.006 (−0.038 to 0.050)	0.786
Zn RDA				
No	Ref		Ref	
Yes	0.092 (0.030–0.153)	0.004	0.122 (0.050–0.193)	0.001
Se	0.006 (−0.029 to 0.041)	0.726	0.028 (−0.005 to 0.062)	0.098
Se RDA				
No	Ref		Ref	
Yes	0.052 (−0.034 to 0.138)	0.232	0.077 (−0.019 to 0.174)	0.114

CI, confidence intervals; Ref, reference; RDA, recommended daily allowance.

Model 1 adjusted demographic characteristics including age, gender, race, and marriage.

Model 2 adjusted all covariates in four groups.

Total femur adjusting age, gender, marriage, PIR, education, BMI, smoking, drinking, physical, CKD, diabetes, dyslipidemia, COPD, cancer, family history of osteoporosis, thiazide, other hypotensor, energy, Na, Ca, caffeine, WBC, and neutrophil.

Femoral neck adjusting age, gender, race, marriage, PIR, BMI, smoking, drinking, physical, CKD, diabetes, COPD, cancer, family history of osteoporosis, thiazide, other hypotensor, energy, Na, and Ca.

### Association between trace elements intake and osteopenia, osteoporosis

The associations between dietary trace element and osteopenia/osteoporosis were shown in Table 4. After adjusting demographic characteristics, dietary copper (OR = 0.604, 95% CI: 0.463–0.788), zinc (OR = 0.651, 95% CI: 0.469–0.854), and selenium (OR = 0.684, 95% CI: 0.479–0.975) intake under RDA levels were related to lower incidence of osteoporosis in elderly hypertensive patients. After adjusting all covariates, dietary copper (OR = 0.581, 95% CI: 0.394–0.858) and zinc (OR = 0.595, 95% CI: 0.429–0.827) intake equal and above RDA levels were associated with increased odds of osteoporosis in elderly hypertensive patients.

**Table 4:** Association between trace element intake and osteopenia and osteoporosis in hypertension.

**Variables**	**Unadjusted**		**Model 1**		**Model 2**	
	**OR (95% CI)**	***P***	**OR (95% CI)**	***P***	**OR (95% CI)**	***P***
**Osteopenia**						
Cu	0.96 (0.90–1.06)	0.585	0.986 (0.897–1.085)	0.773	0.974 (0.887–1.068)	0.566
Cu RDA						
No	Ref		Ref		Ref	
Yes	0.91 (0.75–1.09)	0.279	0.895 (0.729–1.099)	0.284	0.849 (0.698–1.034)	0.102
Fe	1.01 (1.00–1.02)	0.163	1.006 (0.997–1.016)	0.190	1.003 (0.993–1.013)	0.571
Fe RDA						
No	Ref		Ref		Ref	
Yes	1.05 (0.84–1.33)	0.660	1.025 (0.804–1.307)	0.839	1.014 (0.807–1.274)	0.902
Zn	1.01 (1.00–1.02)	0.263	1.007 (0.996–1.019)	0.191	1.005 (0.994–1.017)	0.351
Zn RDA						
No	Ref		Ref		Ref	
Yes	0.95 (0.79–1.13)	0.538	0.927 (0.770–1.116)	0.418	0.946 (0.779–1.147)	0.565
Se	1.00 (1.00–1.00)	0.326	1.000 (0.999–1.002)	0.820	1.000 (0.999–1.002)	0.888
Se RDA						
No	Ref		Ref		Ref	
Yes	0.85 (0.68–1.07)	0.167	0.883 (0.697–1.119)	0.299	0.871 (0.683–1.110)	0.260
**Osteoporosis**						
Cu	0.49 (0.37–0.66)	< 0.001	0.604 (0.463–0.788)	< 0.001	0.581 (0.394–0.858)	0.007
Cu RDA
No	Ref		Ref		Ref	
Yes	0.60 (0.44–0.81)	0.001	0.716 (0.508–1.008)	0.055	0.782 (0.479–1.278)	0.319
Fe	0.98 (0.96–0.99)	0.008	0.989 (0.971–1.008)	0.235	1.001 (0.976–1.026)	0.952
Fe RDA						
No	Ref		Ref		Ref	
Yes	0.68 (0.51–0.89)	0.007	0.744 (0.537–1.030)	0.074	1.012 (0.617–1.662)	0.961
Zn	0.95 (0.92–0.97)	< 0.001	0.969 (0.944–0.994)	0.016	0.965 (0.930–1.000)	0.051
Zn RDA						
No	Ref		Ref		Ref	
Yes	0.66 (0.51–0.86)	0.002	0.651 (0.496–0.854)	0.002	0.595 (0.429–0.827)	0.003
Se	0.99 (0.99–1.00)	< 0.001	0.997 (0.994–1.001)	0.113	0.999 (0.994–1.004)	0.796
Se RDA						
No	Ref		Ref		Ref	
Yes	0.57 (0.41–0.79)	0.001	0.684 (0.479–0.975)	0.036	0.772 (0.489–1.219)	0.260

CI, confidence intervals; Ref, reference; RDA, recommended daily allowance. Unadjusted: Crude model.

Model 1 adjusting demographic characteristics including age, gender, race, and marriage.

Model 2 adjusting all covariates.

Osteopenia adjusting age, race, marriage, BMI, smoking, physical, diabetes, cancer, and hormones.

Osteoporosis adjusting age, gender, race, marriage, PIR, education, BMI, smoking, drinking, physical, CKD, diabetes, dyslipidemia, COPD, cancer, family history of osteoporosis, hormones, thiazide, other hypotensor, energy, Na, and Ca.

## Discussion

Our study investigated the association between dietary trace element intake and osteopenia/osteoporosis in the elderly hypertensive patients. We found dietary copper and zinc intake were positively correlated with BMD on total femur and femoral neck in the elderly hypertensive patients. Higher dietary copper and zinc intakes were associated with lower incidence of osteoporosis in the elderly with hypertension. No statistical significance relationships found between dietary iron, selenium intake and osteopenia/osteoporosis.

Regarding copper intake, we found a positive relationship between copper intake and BMD on total femur and femoral neck. The finding was consistent with the study of Fan et al. (27) who showing a positive association between copper intake and BMD. Copper plays a crucial role in bone metabolism, as it is involved in collagen cross-linking and connective tissue formation (28). Therefore, higher copper intake may contribute to improve bone health, and increasing BMD. Our study also identified a positive association between zinc intake and BMD at the total femur, femoral neck, and intertrochanter. The finding was consistent with the results of Wang et al. (29). Zinc could stimulate osteoblasts and inhibit osteoclasts, thereby promoting bone remodeling. Regarding osteoporosis, our study indicated that higher levels of dietary copper or zinc intake were associated with decreased odds of osteoporosis. The findings were consistent with previous studies reported the beneficial effects of copper and zinc on bone loss (27, 30). Copper has a positive effect on bone metabolism-regulating cells and stimulates the differentiation of mesenchymal stem cells into osteogenic lineage (31). Zinc, on the other hand, acts as a cofactor in enzymatic reactions necessary for bone remodeling and maintenance of bone density (28). Therefore, moderate dietary intake of copper and zinc may help prevent the development of osteopenia and osteoporosis among elderly individuals with hypertension.

The observed associations could be attributed to the specific role of trace elements in bone metabolism. Copper and zinc all participate in important biochemical process that contribute to bone health. Copper is a cofactor for several enzymes involved in the activation of lysyl oxidase, the enzyme responsible for cross-linking collagen fibers to enhance bone strength (28). Furthermore, copper is involved in antioxidant defense, potentially mitigating oxidative stress, which is implicated in bone loss (27). Zinc is not only a component of bone (most of the body's zinc is stored in bones), but also increases osteoblast activity and collagen synthesis while reduces osteoclast-mediated bone resorption (7, 28).

Copper interacts with several other elements in the body, influencing its absorption and utilization. Zinc competes with copper for absorption sites in the intestines, and imbalance in their intake ratios can affect copper metabolism (32, 33). Such interactions highlight the importance of considering overall mineral balance in studies of osteoporosis. Certain medications and medical conditions can also influence copper metabolism and its systemic effects. Chelating agents used in the treatment of Wilson's disease can disrupt copper homeostasis, leading to either deficiency or excess (34). Chronic illnesses such as liver disease may impair copper absorption and utilization, affecting its availability for bone health maintenance (35). Conversely, conditions associated with chronic inflammation may elevate copper levels, influencing its role in bone health through inflammatory pathways (36).

Older hypertensive patients were at higher risk of developing osteopenia and osteoporosis. Increased intake of copper and zinc through diet or supplementation may be beneficial for bone health in hypertensive patients. Additionally, exploring the optimal supplemental dose of each trace element will help reduce the incidence of osteopenia or osteoporosis in elderly hypertensive patients.

Some limitations should also be acknowledged for our study. First, dietary copper intake primarily comes from foods such as nuts, seeds, whole grains, shellfish, and organ meats (37). However, non-dietary sources like copper utensils, pipes in area with older infrastructure or from drinking water in regions with high copper content can also contribute to overall copper exposure (38). This variability underscores the challenge in accurately assessing total copper intake through 24-h dietary recall used. However, some studies have shown that daily dietary intake may be sufficient to be evaluated based on two 24 h dietary recalls (39–41). In addition, NHANES is a cross-sectional survey project, and all data were obtained at a single time point, making it difficult to establish temporal relations and causality. Finally, a few covariates were adjusted, but residual confounding by unmeasured factors cannot be eliminated.

## Conclusion

Higher dietary copper and zinc intakes were associated with lower incidence of osteoporosis in the elderly with hypertension. The study emphasizes the significant role of dietary trace element in osteoporosis among elderly individuals with hypertension. To enhance or sustain bone mass and mitigate the risk of osteoporosis, clinical interventions should consider appropriate augmentation of dietary trace element intake, without excess.

## Data availability statement

Publicly available datasets were analyzed in this study. This data can be found here: NHANES database, https://www.cdc.gov/nchs/nhanes.

## Ethics statement

The studies were conducted in accordance with the local legislation and institutional requirements. The participants provided their written informed consent to participate in this study.

## Author contributions

MC: Conceptualization, Project administration, Supervision, Writing – original draft, Writing – review & editing. LJ: Data curation, Formal analysis, Investigation, Methodology, Writing – review & editing. RG: Data curation, Formal analysis, Investigation, Methodology, Writing – review & editing.
